# Notes and News

**Published:** 1903-05-16

**Authors:** 


### Notes and News.

### HOSPITAL MEETINGS AND FESTIVALS.

Victoria Hospital for Children, Chelsea.—Annual Meeting, Wednesday, May 20.

Metropolitan Hospital.—Festival Dinner, Wednesday, May 20, 7 p.m., at the Whitehall Rooms, Hotel Metropole.

Mount Vernon Hospital for Consumption.—Festival Dinner, Wednesday, May 20, 7 p.m., at Hotel Cecil.

Alexandra Hospital.—Annual Meeting, Thursday, May 21.

Hospital for Sick Children, Great Ormond Street.—Annual Meeting, Monday, May 25, at 5 p.m.

North-Eastern Hospital for Children.—Annual Meeting, Thursday, May 28, at 4.30 p.m.

Hospital for Consumption, Brompton.—Annual Meeting, Thursday, May 28, at 4 p.m.

A grand bazaar in aid of the Royal Free Hospital is being arranged by Princess Christian of Schleswig-Holstein, president of the hospital, and Princess Louise Augusta of Schleswig-Holstein, president of the Ladies' Association, to take place at Prince's Skating Club, Knightsbridge, on Wednesday, June 10th, and two following days.

At a meeting of the Childhood Society on May 11th, at 7 St. James's Square, S.W., Professor J. Edgar, M.A., Professor of Education in the University of St. Andrews, delivered an address on "The Universities and Scientific Study of Children with Special Reference to the Teaching Profession." Subsequently the following resolution, proposed by T. W. Brabrook, C.B., and seconded by Sir James Crichton Browne, M.D., F.R.S., was carried unanimously:—"That it is desirable that the Elementary Education (of Defective and Epileptic Children) Act, 1899, should be compulsory on educational authorities, and that special training should be given at one or more training colleges for teachers who wish to qualify for the care of defective children. That the Government be requested to institute an inquiry into the best means of dealing with defective classes in after life and for determining the authority to be entrusted with that duty."

The annual meeting of the Chelsea Hospital for Women will be held on Thursday, May 21st, at 4 p.m., the Chairman, the Right Hon. the Lord Glenesk, presiding.

The ball which is being arranged by the Countess of Derby in aid of the London Hospital is evidently going to be a great success. The demand for tickets is so great that it has been found necessary to move the offices of the ball from the hon. treasurer's (Mr. Garratt) private residence to the Social Bureau, 30 New Bond Street, where all particulars can now be obtained. The ball is to take place on June 9th, at the Albert Hall.

The Fishery Board of Scotland has issued a paper on the cause of salmon disease. The paper is by Mr. J. H. Patterson, of Glasgow, and the results of his investigations are of considerable interest and importance. He shows that salmon disease is not due to the fungus saprolegnia ferax, as has been supposed, but is caused by a specific micro-organism, bacillus salmonis pestis. As is well known the saprolegnia fungus does not grow in salt water, and is not met with in fish fresh run from the sea, whereas the bacillus described by Mr. Patterson grows well in both salt and fresh water. A cold temperature is favourable to the growth of the micro-organism. Fish which have died from the disease, as well as living fish which are affected, can spread the contagion to others in the same waters, and therefore all dead fish which are found should be removed and burned. The salmon tribe are the most susceptible to the disease, but other genera are not immune. It is to be hoped that now the cause has been recognised some method of coping with this destructive fish pestilence may be discovered.

The excellent work which is being done among the men of the British Navy by Miss Weston and Miss Wintz is too well appreciated to need any commendation. At the annual meeting held at Exeter Hall on the 5th inst. Miss Weston showed how rapidly the work had been increasing in every direction. The most important feature in the year's work as indicating progress and prosperity was the pulling down of old buildings at Portsmouth and the commencement of a very handsome pile of buildings for the enlargement of the Royal Sailors' Rest at Devonport, to be called "The Victoria Memorial Block." To this block both the King and Queen Alexandra had given cabins. Its cost would be about £15,000. Miss Weston had during the year signed the first contract for £9,190. Funds and cabins were needed for this building. Miss Wintz, in her interesting account of the working of the commissariat at the Royal Sailors' Rests, Portsmouth and Devonport, which are under her supervision, said that the receipts taken over the counters totalled £25,035. The sleepers during the twelve months were 290,123, an increase of 40,223 on last year. These figures alone are sufficient to indicate what an immense amount of good is being done each year by the Royal Sailors' Rests, and it is to be heartily desired that Miss Weston and Miss Wintz will receive all the help they require in carrying on these excellent institutions.

